# Experimental and digital investigations of heterogeneity in lower cretaceous carbonate reservoir using fractal and multifractal concepts

**DOI:** 10.1038/s41598-023-47681-w

**Published:** 2023-11-20

**Authors:** Mohamed Soufiane Jouini, Abdulquadri O. Alabere, Mohammad Alsuwaidi, Sadoon Morad, Fateh Bouchaala, Osama A. Al Jallad

**Affiliations:** 1 Department of Mathematics, Khalifa University, P.O. Box 127788, Abu Dhabi, United Arab Emirates; 2 Department of Petroleum Geosciences, Khalifa University, P.O. Box 127788, Abu Dhabi, United Arab Emirates; 3 Halliburton Service in the Middle East and Asia Pacific Areas, P.O. Box 57, Abu Dhabi, United Arab Emirates; 4 BP Exploration, Chertsey Road, Sunbury-On-Thames, Middlesex, TW16 7LN UK; 5 Sukhoor Consulting Company, Abu Dhabi, United Arab Emirates

**Keywords:** Geology, Applied mathematics

## Abstract

Characterization and prediction of reservoir heterogeneity are crucial for hydrocarbon production. This study applies the multifractal theory using both numerical and experimental data to characterize quantitatively the heterogeneity of pore structures in Lower Cretaceous limestone reservoir from the United Arab Emirates. Fractal dimensions calculated from three dimensional digital images showed good correlation (R^2^ =  + 0.69) with experimental high-pressure mercury injection (HPMI) measurements. Moreover, both experimental and numerical fractal dimensions correlate well with experimental HPMI porosity measurements. Multifractal parameters such as the non-uniformity degree of the pore structures *Δα*, the asymmetry degree in the vertical axis *Δf(α)*, the concentration of pore size distribution *α*_*0*_ and the asymmetry degree in the horizontal axis *R*_*d*_ estimated from digital and experimental data correlated well and revealed ability to quantitatively describe samples heterogeneity. The ranges of digital and experimental multifractal parameters provided the means to differentiate between homogeneous and heterogeneous samples.

## Introduction

Carbonate reservoir rocks are highly heterogeneous and reveal complex pore geometry at various scales due to diagenesis and depositional processes. Pore structure is commonly characterized by the size, volume, connectivity, shape and distributions of pore radii and pore throats^1,2^. The concept of fractal dimension was introduced to describe the irregularity and complexity of structures, utilizing the idea of self-similarity in an object (Mandelbrot, 1974, 1977, 1983). Several studies investigated sandstone, shale, and carbonate reservoir rocks suggesting that they reveal fractal behaviour within a certain range of length^3–7^. The fractal dimension is a real number ranging from 1 to 2 for 2-D objects and from 2 to 3 for 3-D objects^8^. The main advantage of utilizing fractal theory is its ability to connect microscopic geometry patterns with macroscopic structural properties and it has been largely applied in geosciences to investigate carbonates pore space structures based on various experimental and digital imaging approaches. The concept of multifractals goes beyond the standard dimensions of fractals and can explain the hidden information that is not accounted by the conventional dimensions of fractals^9,10^. Several studies have applied fractal and multifractal theories using High Pressure Mercury Injection (HPMI) experimental measurements to investigate the irregularity of porous media and its impact on various rock properties such as permeability, pore throat diameter and porosity^11–19^. In the literature, several models relating capillary pressure and saturation introduced fractal dimension to consider geometric characteristics of the pore space^11,20,21^. A universal capillary pressure model was developed by Li and Horne^11^ to characterize heterogeneity in carbonate samples from the Geysers geothermal field by matching the capillary pressure model to the experimental data using experimental fractal dimension. Zhang et al.^15^investigated the heterogeneity in Lower Carboniferous carbonate reservoirs in the Marsel area in Kazakhstan using fractal dimensions computed from HPMI data and found relatively good correlations of fractal dimensions with several rock properties such as porosity, permeability, sorting coefficient, and skewness. Guan et al.^22^ and Zhang et al.^23^ implemented multifractal analysis based on HPMI experimental measurements to investigate samples heterogeneity using multifractals parameters such as the non-uniformity degree and the width of singularity spectra and found good correlations between the multifractals parameters and the heterogeneity degree of pore size distributions in studied samples.

Fractal and multifractal theories were implemented in other studies using digital images of reservoir samples^24–31^. Technological advancements in 2D and 3D image acquisition systems allow exploring pore space at several length scales from millimetre to nanometre using 3D X-ray Micro-Computed Tomography (3D-MCT), Nano-Computed Tomography (NCT), and 2D Scanning Electron Microscopy (SEM). Following the image acquisition, the pore space is extracted using image segmentation methods to derive segmented binary images used to reveal the geometric distribution of pores^32^. Subsequently, techniques such as box-counting and gliding box methods can be applied on these images to estimate the fractal dimension^33^. Jouini et al.^26^ investigated the behavior of multifractal dimensions in SEM images of carbonate reservoir at several length scales and showed that fractal dimension concept can be used for a quantitative characterization of pore space heterogeneity. Following this study, Vega and Jouini^27^ proposed an analytical model to upscale porosity using the numerical fractal dimension values from SEM and thin section images of carbonate samples. Jouini et al.^30^ investigated heterogeneity at the pore scale for twenty rock samples from sandstone and carbonate reservoirs using multifractal theory based on 3D X-ray micro-computed tomography images. Authors showed that the capacity dimension D_0_ and the information dimension D_1_ correlate with porosity and permeability simulated from images, respectively. In addition, Jouini et al.^30^ showed the ability of multifractal parameters to classify groups of rock samples according to their degree of heterogeneity.

Few studies have applied both numerical and experimental fractal concepts to analyse pore structures in clastic and carbonate reservoirs. Rahner et al.^34^ established a correlation between image and experimental fractal dimensions in relatively homogeneous shale and tight gas sandstones using 3D MCT and NCT images. Chen et al.^35^ derived experimental fractal dimensions from HPMI data of six sandstone reservoirs and compared them with numerical fractal dimension calculated from 2-D SEM and 3-D MCT images. The fractal dimensions from 2-D and 3-D images were combined in a single parameter using a bridge function and results show that they are consistent with experimental fractal dimensions for only simple pore structure but not for more complex pore structures. Recently, Shi et al.^36^ calculated both experimental and digital fractal dimensions from HPMI and SEM data, respectively, in Lower carboniferous carbonate reservoir without discussing the relationship between image and experimental fractals due to the absence of representability between HPMI and SEM length scales. In addition to their scarcity, these studies did not investigate the relationship between experimental and digital multifractal parameters.

In this study, we investigated the pore structure distribution and heterogeneity of Lower Cretaceous carbonate reservoir from Abu Dhabi, United Arab Emirates (UAE) using coupled quantitative descriptors obtained from multifractal theory based on three dimensional digital images and HPMI experimental measurements. Furthermore, petrophysical properties were simulated numerically at pore scale to elucidate their correlation with multifractal descriptors. This study seeks to address the gap in analysing pore structure heterogeneity using the multifractal concept by applying the theory to both experimental and digital data.

## Material and methods

### Geological setting

The Thamama Group (Barriasian-Aptian) is a carbonate reservoir that was deposited in shallow-marine, low-energy carbonate ramp and contains four formations: Habshan, Lekhwair, Kharaib and Shuaiba. Data from this study comes from Lekhwair Formation specifically from the Upper Thamama Zone D. The main depositional facies of Lekhwair Formation include high-frequency coarsening upward cycles of shallow-subtidal skeletal-Bacinella floatstones, skeletal-peloidal wackestones; mud-dominated packstones, capped by skeletal-ooidal grainstones^37^. The carbonate samples were selected carefully to represent six different reservoir rock textures defined within the Upper Thamama Zone D. The Upper Thamama Zone D frequently is considered as low resistivity pay zone (LRPZ) due to the presence of multimodal pore network^37^.

### Samples and experiments

**Figure 1:**
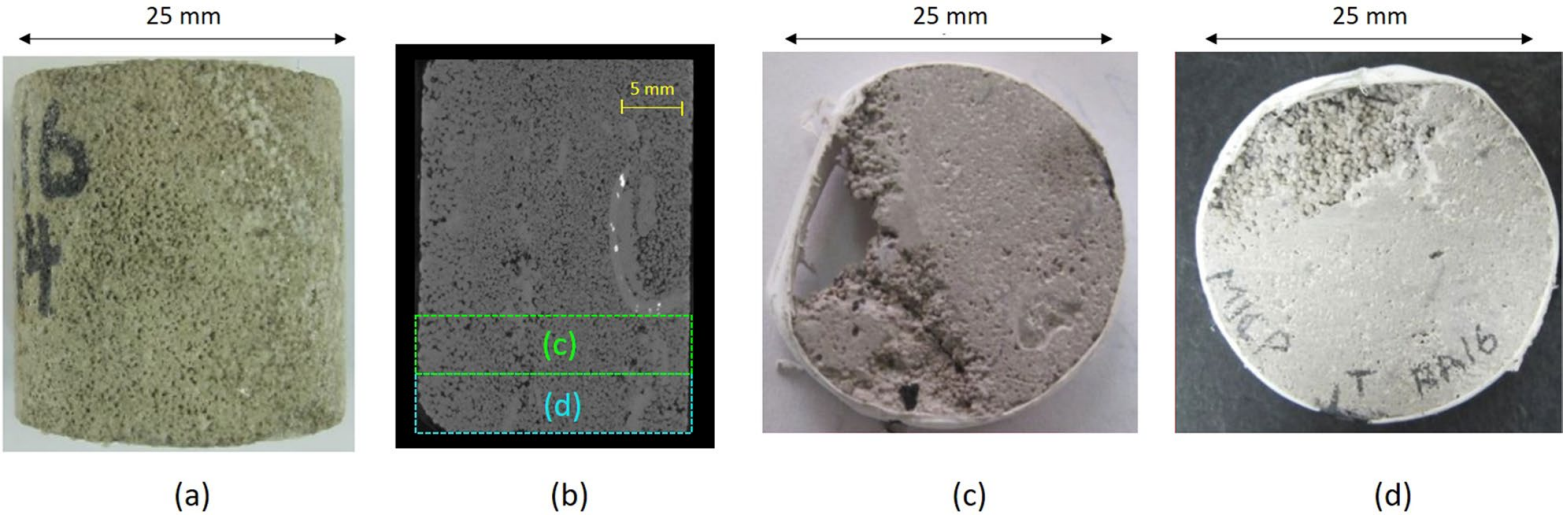
HPMI experiments subsample selection and image acquisition at high resolution for Sample S_1_: (**a**) core plug photograph, (**b**) X-Ray 3D-MCT at 20 μm resolution and selection by visual inspection of subsamples, (**c**) Subsample (trim) to be examined at high resolution, and (**d**) Subsample used for HPMI measurements.

**Figure 2:**
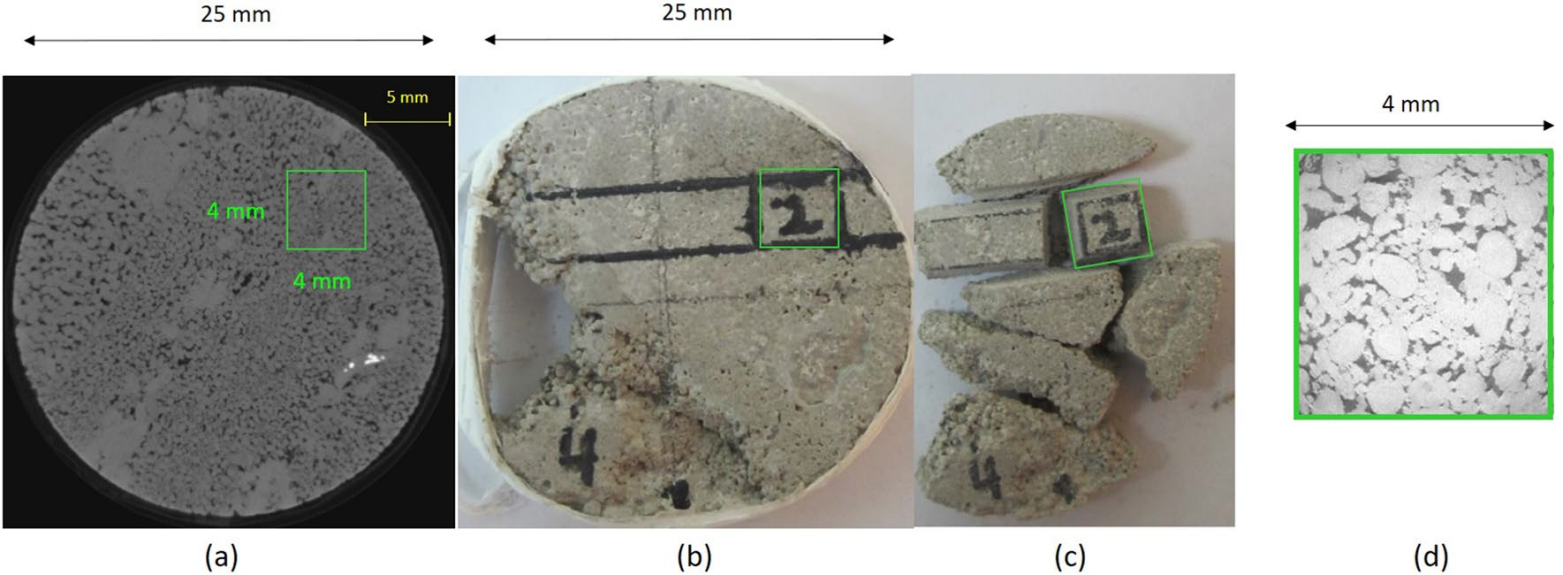
Subset selection and image acquisition at high resolution for Sample S_1_: (**a**) Location of 4 mm^3^ extracted subset on 3D X-Ray MCT at 20 μm resolution, (**b**) subset location on real core plug, (**c**) Subset extraction, and (**d**) Subset scanned at 4 μm resolution.

Samples used for this study include six core plugs S_1_–S_6_ of 25 mm in diameter from Lower Cretaceous, shallow marine limestone reservoir in the UAE covering different depths and textural characteristics. The selected limestone samples are composed of allochems dominated by skeletal fragments, ooids, peloids, and oncoids (Table 1). The limestones exhibit multi-modal pore network consisting of macropore (e.g., intergranular, moldic, vuggy pores) and micropores within skeletal fragments and between micrite particles, the distribution of which is controlled by depositional textures and diagenetic alterations^30,37^. X-Ray MCT and NCT are non-invasive acquisition methods used to reconstruct 3D tomographic image models of rock^38^. These systems consist of X-ray emitting source and detector receiving the attenuated X-Ray signal crossing the sample. To generate the 3D image, a series of acquisitions were obtained at different angles. Projected data are reconstructed numerically to generate the 3D grey level image representing the sample. The attenuation of incident X-rays is related to the sample inner density. Thus, high grey levels denote the solid phase whereas low grey levels are pores. To investigate the pore geometries and grain morphologies, a segmentation procedure needs to be implemented to separate solid and porous phases. The samples were scanned at coarse scale (20 μm resolution) using a Zeiss Xradia X-ray micro-computed tomography scanner (Fig. 1). Subsequently, two 5 mm high subsamples representing the same sample texture were extracted physically from each core plug by visual inspection of the 3D images (Fig. 2).

**Table 1:** Brief description of the different samples, core plug features and their limestone textures.

Sample	Limestone Dunham texture
S_1_	Skeletal ooidal packstone to grainstone
S_2_	Skeletal oncoidal grainstone to rudstone
S_3_	Skeletal-peloidal packstone
S_4_	Oncoidal packstone
S_5_	Skeletal wackestone to mud-dominated packstone
S_6_	Oncoidal wackestone to floatstone

**Figure 3:**
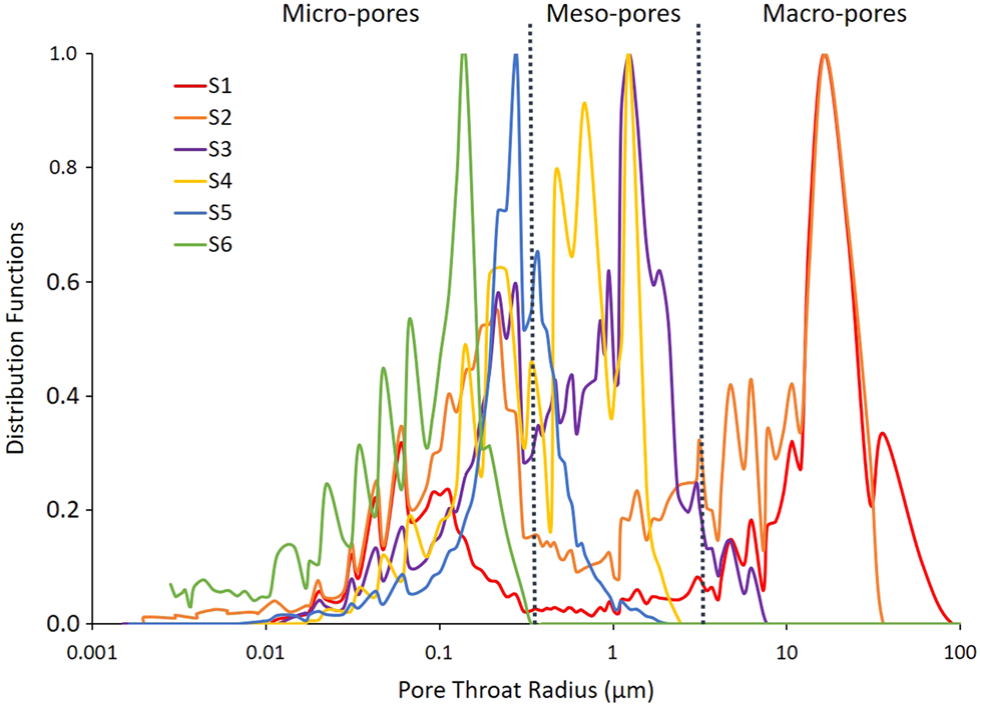
Semi logarithmic plot of experimental HPMI Pore throat distributions of the six samples.

**Figure 4:**
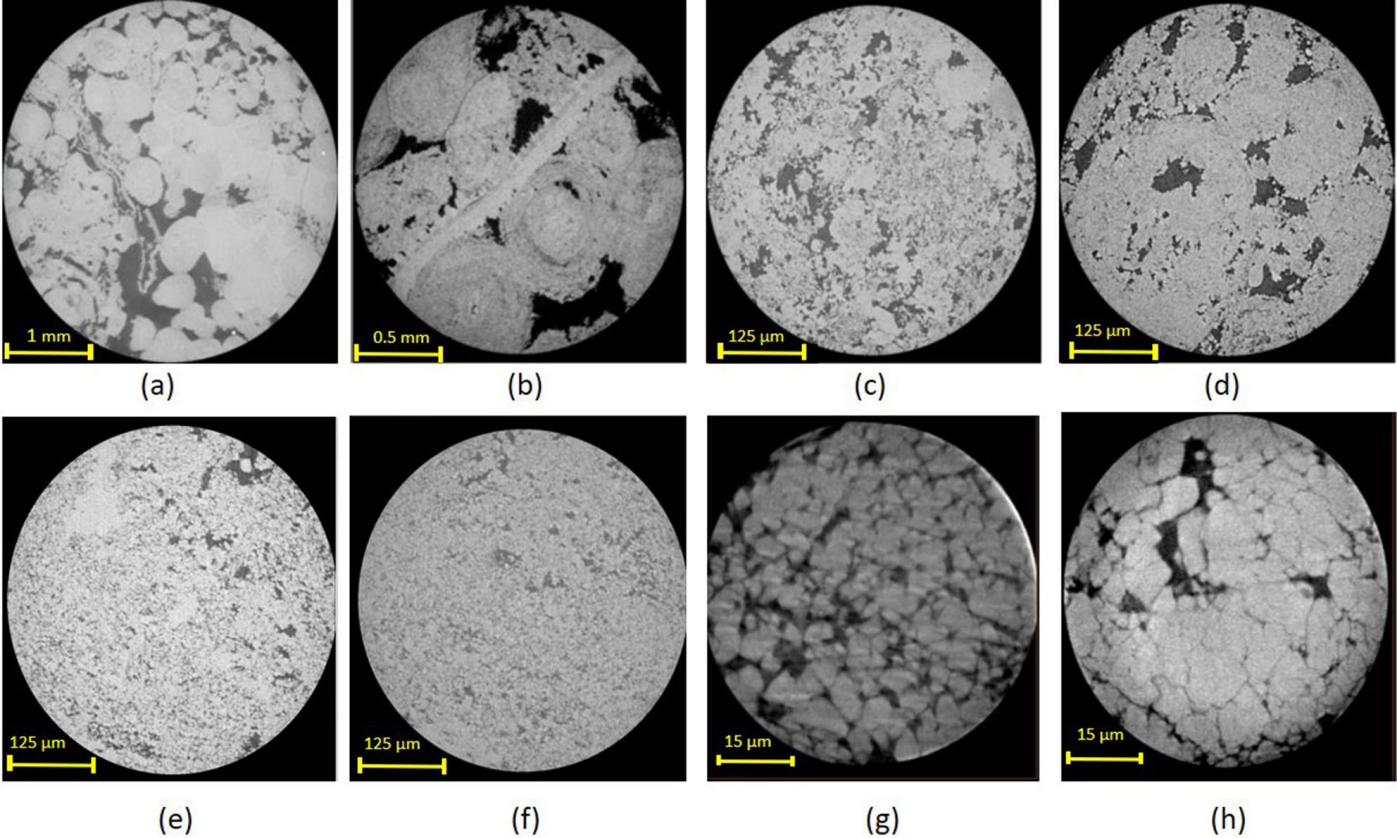
Horizontal slices obtained from the six samples (extracted from 3D images): (**a**) 3D-MCT for sample S_1_ at 2 µm resolution, (**b**) 3D-MCT for samples S_2_ at 1 µm resolution, (**c**–**f**) 3D-MCT for samples S_3_ to S_6_ at 0.5 µm resolution, and (**g**,**h**) 3D-NCT for samples S_5_ and S_6_ at 60 ηm resolution.

**Figure 5:**
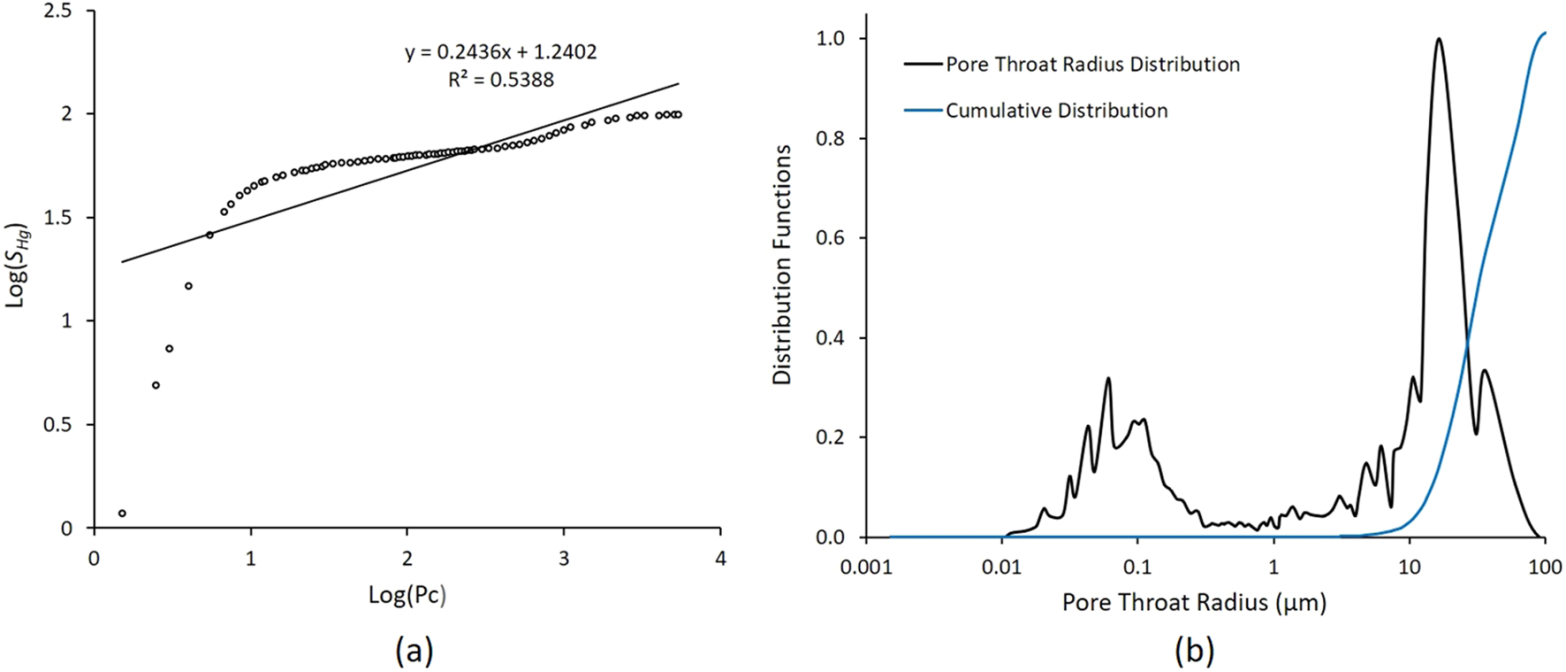
(**a**) Log–log plot relating *S*_*Hg*_ the mercury saturation to the capillary pressure *P*_*c*_ for the sample *S*_*1*_, and (**b**) Plot of pore throat radius distribution and its cumulative function.

The first subsample was used for HPMI measurements to experimentally characterize the capillary pressure curves. This approach involves introducing mercury into samples at increasing pressure, ranging from 0.5 psi to 60,000 psi. The analysis of mercury intrusion volume at various pressures allows for the derivation of a distribution function for pore throat sizes, along with determining the permeability and porosity of the subsample. This experimental method is valuable for rock typing, analysis and interpretation of core data. The results of the pore-throat distributions have yielded suitable information, allowing for the selection of the optimal scanning resolution that effectively captures the majority of pore features. The best resolution that X-ray MCT systems can reach depends on the energy and detector size. As the field of view is constrained by the detector size, smaller subsets were extracted physically from subsamples and scanned at higher resolutions to capture most pore features based on HPMI experimental results (Fig. 3). Furthermore, when experimental results reveal that most pore throats were below the micron, then NCT was implemented to capture pore network. For instance, the 0.5 μm resolution scan of sample S_5_ revealed few inter-particle pores that were not connected at this resolution (Fig. 4). Furthermore, the pore throat distribution curve of the same sample confirmed the presence of most pores below the 0.5 μm resolution (Fig. 5). Therefore, smaller representative subsets were further extracted and scanned using NCT at a resolution of 60 ηm per voxel as illustrated in Fig. 4h. Table 2 summarizes dimensions and resolutions of X-Ray MCT and NCT scanned data for the six studied samples.

**Table 2:** Subset dimensions and corresponding 3D-MCT and NCT image acquisition resolutions.

Samples	Micro-CT		Nano-CT	
	Dimensions (mm^3^)	Resolution (µm)	Dimensions (µm^3^)	Resolution (ηm)
S_1_	64	4	NA	NA
S_2_	8	2	NA	NA
S_3_	0.125	0.5	NA	NA
S_4_	0.125	0.5	NA	NA
S_5_	0.125	0.5	216	60
S_6_	0.125	0.5	216	60

### Application of fractal and multifractal theories for HPMI data

#### Application of fractal theory for HPMI

HPMI measurements are extremely valuable because they: (i) represent valuable experimental information to characterize the distribution of fluids within the reservoir and rock transport properties, and (ii) provide capillary pressure curves that are commonly used to infer pore size distributions of rock samples. The capillary pressure and saturation relationship depends on several parameters including pore throats size distribution, grain and pore geometry. Depending on the implemented model, the calculated fractal dimensions may reflect specific characteristics of the pores. For example, Li et al.^11^ and Li^21^ developed a model based on a power-law function as in the following equation:

1 $$N\left(r\right)=b {r}^{-{D}_{f}}$$

where* r* is the radius to fill a unit of the fractal object, *N(r)*is the number of the units having a radius *r* needed to fill the whole fractal object, *b* is a factor of proportionality and *D*_*f*_ is the fractal dimension. The model assumes that a pore is represented by capillary tube with a length of *l* and a volume equal to *πlr*^*2*^. The number of units *N(r)* can be calculated based on experimental capillary pressure curves measurements. Furthermore, a common assumption implemented in the model considers *l* independent of *r*. When the pore structure of the rock is fractal, *D*_*f*_ the fractal dimension can be determined by finding the relationship between *S*_*Hg*_ the mercury saturation and *P*_*c*_ the capillary pressure as the following equation:

2 $$\mathrm{log}\left({S}_{Hg}\right)=\left({D}_{f}-2\right)\mathrm{log}\left({P}_{c}\right)+\mathrm{log}\left(a\right)$$

This fractal dimension reflects the pore size distribution^11^. Moreover, the estimated fractal dimension model revealed high correlations with petrophysical properties of core plugs^11,39^. Other models can provide fractal dimension reflecting the characteristics of pore volume distributions in three dimension. Friesen et al.^20^ implemented a model describing measurements of the pore volume of a number of coal and char samples by mercury intrusion porosimetry. The fractal dimension was determined from the relationship in the following equation:

3 $$\mathrm{log}\left(\frac{{dS}_{Hg}}{{dP}_{c}}\right)=\left({D}_{f}-4\right)\mathrm{log}\left({P}_{c}\right)+\mathrm{log}\left(b\right)$$

where *b* is a constant.

In addition, some models can provide a fractal dimension characterizing the roughness of the pore surface Zhang and Li^40^. In our study, we focused on the model proposed by Li^21^ as one of our goals is to find the correlation between fractal dimension and petrophysical properties such as porosity and permeability in the samples.

**Figure 6:**
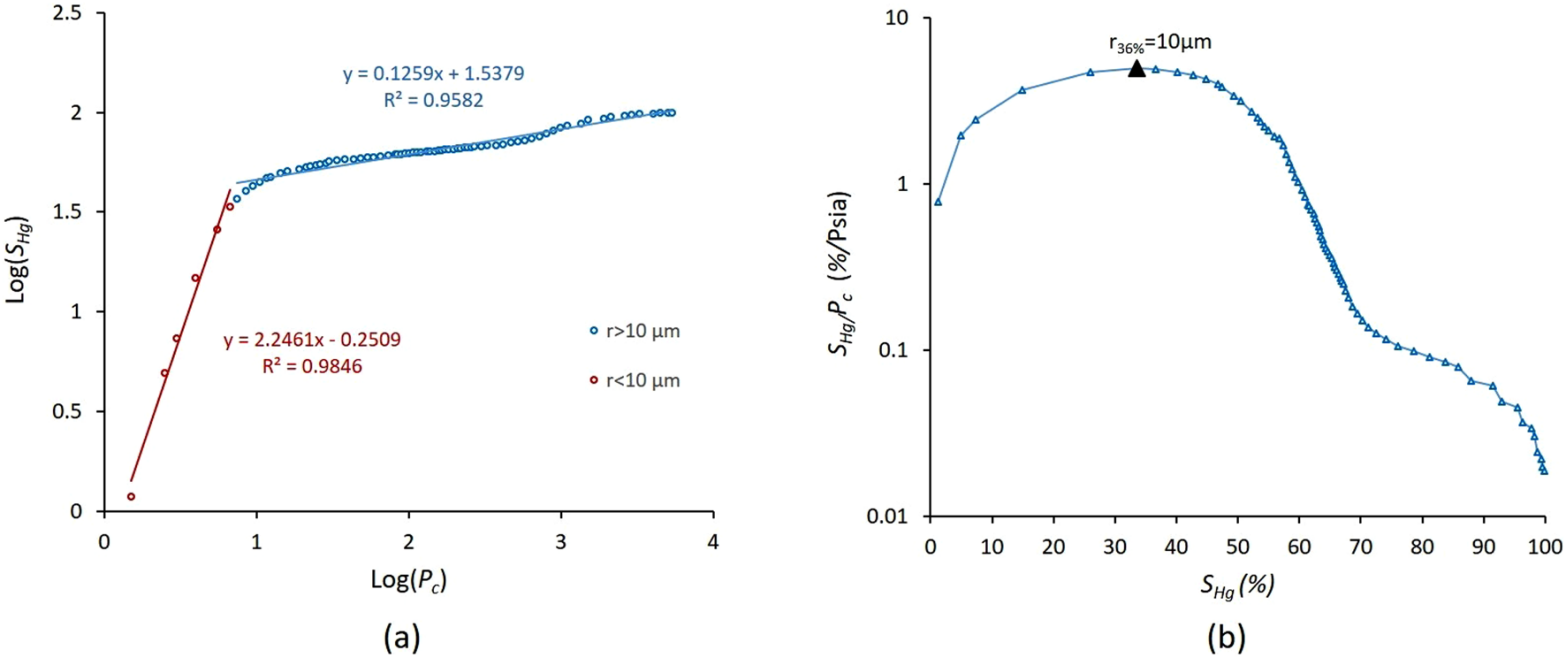
(**a**) Log–log plot relating *S*_*Hg*_ the mercury saturation to the capillary pressure *P*_*c*_ for the sample *S*_*1*_ illustrates the double-fractal characteristics, and (**b**) Plot of log $\frac{{S}_{Hg}}{{P}_{c}}$ versus ${S}_{Hg}$ illustrating the position of Swanson’s point: pore throat radius r_36%_ = 10 μm for the sample *S*_*1*_.

Based on Eq. (1) the log − log plot of *S*_*Hg*_ and* P*_*c*_ was obtained from HPMI experimental measurements for the Sample S_1_ as illustrated in Fig. 5a. The pore throat radius distribution revealed in Fig. 5b abimodal behaviour and the cumulative distribution function showed a dramatic increase at pore throat size of r_36%_ = 10 μm corresponding to the value log(*P*_c_) = 0.87 PSI at *S*_*Hg*_ = 36%. Using (2), the fractal dimension of pores *D*_*f*_ was estimated as 2.24. The plot revealed a poor linear relationship between the variables with a coefficient of determination R^2^ =  + 0.53. However, the curve showed a double-fractal characteristic depending on the range of the capillary pressure *P*_*c*_. Figure 6a illustrates a strong linear relationship between *S*_*Hg*_ and *P*_*c*_ with a coefficient of determination R^2^ =  + 0.98 for all values less than log (*P*_*c*_) = 0.87 PSI. The same strong relationship is observed for the values larger than log (*P*_*c*_) =  + 0.87 PSI with a coefficient of determination R^2^ =  + 0.95. Similar result was observed in several previous studies when deriving fractal dimension *D*_*f*_ from HPMI experimental measurements Lai and Wang^41^.

Moreover, many studies employed multivariate statistical analysis revealed that porosity and permeability showed better results when correlated to pore radius *r*_*p*_ where *p* is the percentage of saturation of the non-wetting phase^42,43^. Therefore, the Swanson’s method was implemented to determine the segmentation point for the fractal dimension from each HPMI curve for the studied samples^44^. Several researchers used the position of the maximum value of the plot of *S*_*Hg*_/(*P*_*c*_) versus *S*_*Hg*_, which is known as the Swanson point, to find the transition between highly and poorly connected pores from HPMI curves^42,43^. The triangular purple point represents the Swanson point of the sample S_1_ (Fig. 6b). Based on the Swanson’s point, the fractal dimension of the small pores *D*_*S*_ and the large pores *D*_*L*_ of sample S_1_ were estimated from the slopes using Eq. (9) respectively as *D*_*S*_ = 4.24 and *D*_*L*_ = 2.12. This result indicates that the fractal dimension *D*_*S*_ = 4.24 of sample S_1_ is greater than 3.0 contradicting the Euclidean dimension. Therefore, this range of pores has a low impact on pore structure evaluation. The fractal dimension of large pore throats *D*_*L*_ = 2.12 which is conformed to the Euclidean dimension.

#### Application of multifractal theory for HPMI

The Box-Counting (BC) method is a standard technique used to estimate multifractal parameters revealing the complexity of a data. This method analyses patterns at several length scales to capture self-similarities by zooming in and out in the data. Furthermore, the technique requires the measurements to be equally spaced with a scale of ε. In our study, HPMI measurements were irregularly spaced so linear interpolation were used to obtain regularly spaced data points divided into *N* = 2^*10*^ = 1024 sub-intervals. Practically, for each scale ε, the mass probability function P_k_(ε) of the k^th^ interval is defined as the ratio between *N*_*i*_*(ε)* the pore volume of a *k*^th^ interval and *N*_*t*_ the total pore volume. The mass probability function is an exponential function of scale *ε* as defined as in the following equation:

4 $${P}_{k}(\upvarepsilon )\sim {\upvarepsilon }^{{\alpha }_{k}}$$

where ${\alpha }_{k}$ is the singularity index.

Noted that the number of boxes *N(ε)* increases exponentially with the scale *ε* as in the following equation:

5 $${N}_{\alpha }(\upvarepsilon )\sim {\upvarepsilon }^{-f(\alpha )}$$

where *N*_α_(*ε*) represents the number of boxes with singular strength in the interval [*α*, *α* + d*α*] and *f(α)* is the singularity spectrum^12^.

The generalized dimensions *D*_*q*_ and the partition function *χ(q,ε)* are defined for *q* > *1* as in the following equation:

6 $${D}_{q}=\frac{1}{q-1}\underset{\varepsilon \to 0}{\mathit{lim}}\frac{log{\sum }_{k}{P}_{k}^{q}(\varepsilon ) }{log\varepsilon }$$

where $$\chi \left(q,\varepsilon \right)={\sum }_{j}{P}_{j}^{q}(\varepsilon )$$.

For *q* = *1, D*_*1*_ is expressed as in the following equation:

7 $${D}_{1}=\underset{\varepsilon \to 0}{\mathit{lim}}\frac{{\sum }_{k}{P}_{k}(\varepsilon )log{P}_{k}(\varepsilon ) }{log\varepsilon }$$

The mass exponent *τ*_*q*_ and *D*_*q*_ the generalized dimension are related through the following equation:

8 $${\tau }_{q}=(q-1){D}_{q}$$

For homogeneous objects the mass exponent *τ*(*q*) reveals a linear relationship with moments *q*. Conversely, for heterogeneous objects, the slope of *τ*(*q*) may change with respect to *q* and the deviation is related to the degree of the heterogeneity.

In addition, the multifractal theory provides a relationship between the singularity spectrum *f*(*α*) and the singularity *α* as in as in the following equations:

9 $$f\left(\alpha \right)=q\alpha -{\tau }_{q}$$

10 $$\alpha =\frac{d{\tau }_{q}}{dq}$$

**Figure 7:**
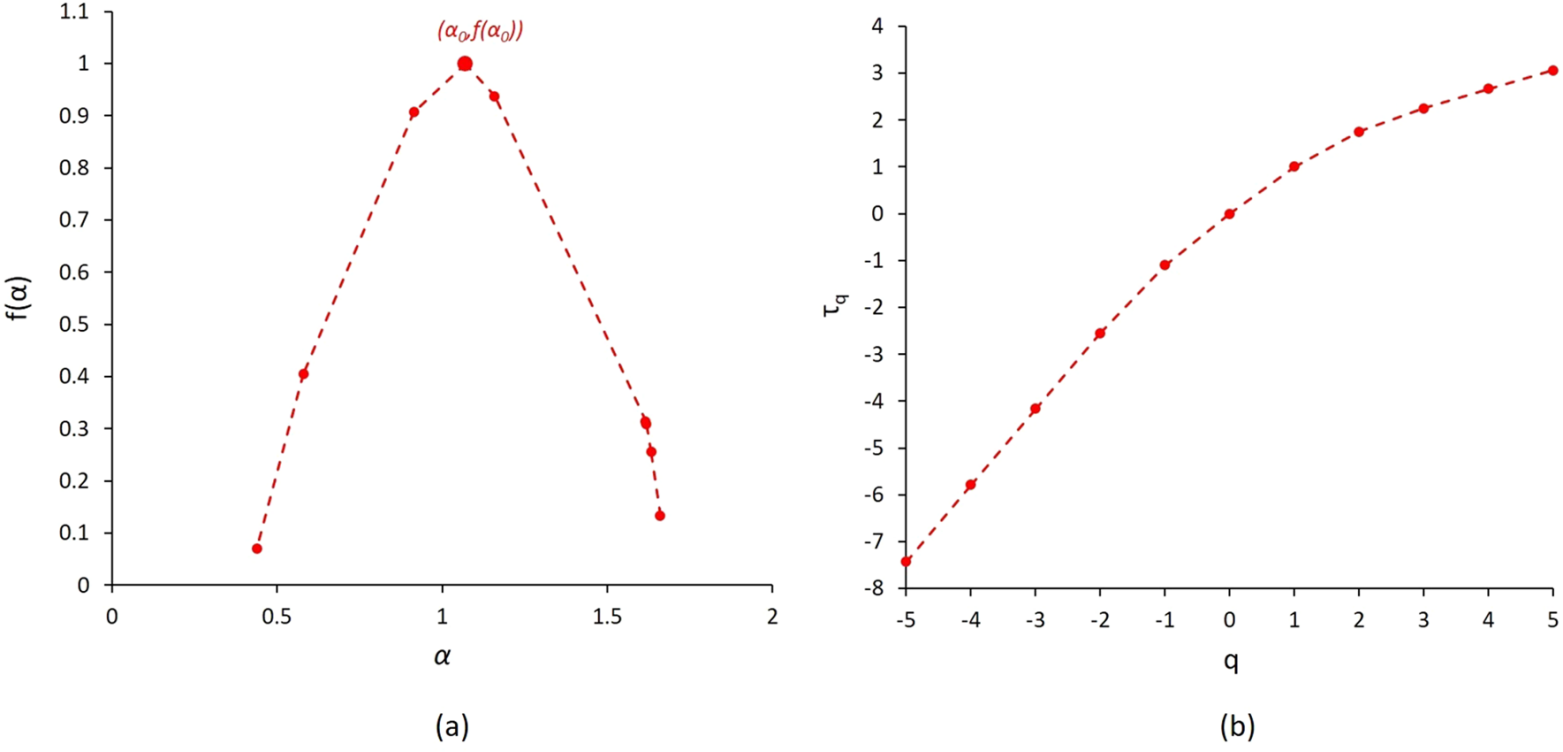
Sample S_1_: (**a**) singularity spectrum f(α) calculated from HMPI experimental measurements, and (**b**) the mass exponent τ_q_.

Figure 7 illustrates the mass exponent and singularity spectrum curves for sample S_1_.

The variable α_0_ represents the concentration of pore size distribution corresponding to the maximum value of the singularity spectrum as illustrated in Fig. 7. The parameter *Δα* = *α*_*min*_-*α*_*max*_ denotes the non-uniformity degree of the pore structures analysed, the larger *Δα* the higher the data spatial complexity, where *α* belongs to the interval [α_min_ α_max_]. The symmetry of the singularity spectrum curve *f*(*α*) helps for a quantitative assessment of data heterogeneity. Indeed, uniform data are characterized by symmetric curves whereas heterogeneous ones reveal curve asymmetry^9,45^. The parameter *Δf* = *f*(*α*_*min*_) − *f*(*α*_*max*_) denotes the shape characteristics of singularity spectra. The curve *f*(*α*) reveals an asymmetry to the right or to the left according to the dominant probability subset. *R*_*d*_ = (*α*_*0*_-*α*_*max*_)-(*α*_*0*_-*α*_*min*_) represents the asymmetry degree in the horizontal axis of the range [*α*_*min*_*, α*_*max*_] with respect to *α*_*0*_. When the value of *R*_*d*_ is positive, it indicates that the porosity distribution is mostly in sparse areas, and the opposite is true for negative values^46^.

### Image analysis and simulations of rock properties

#### Image Segmentation

**Figure 8:**
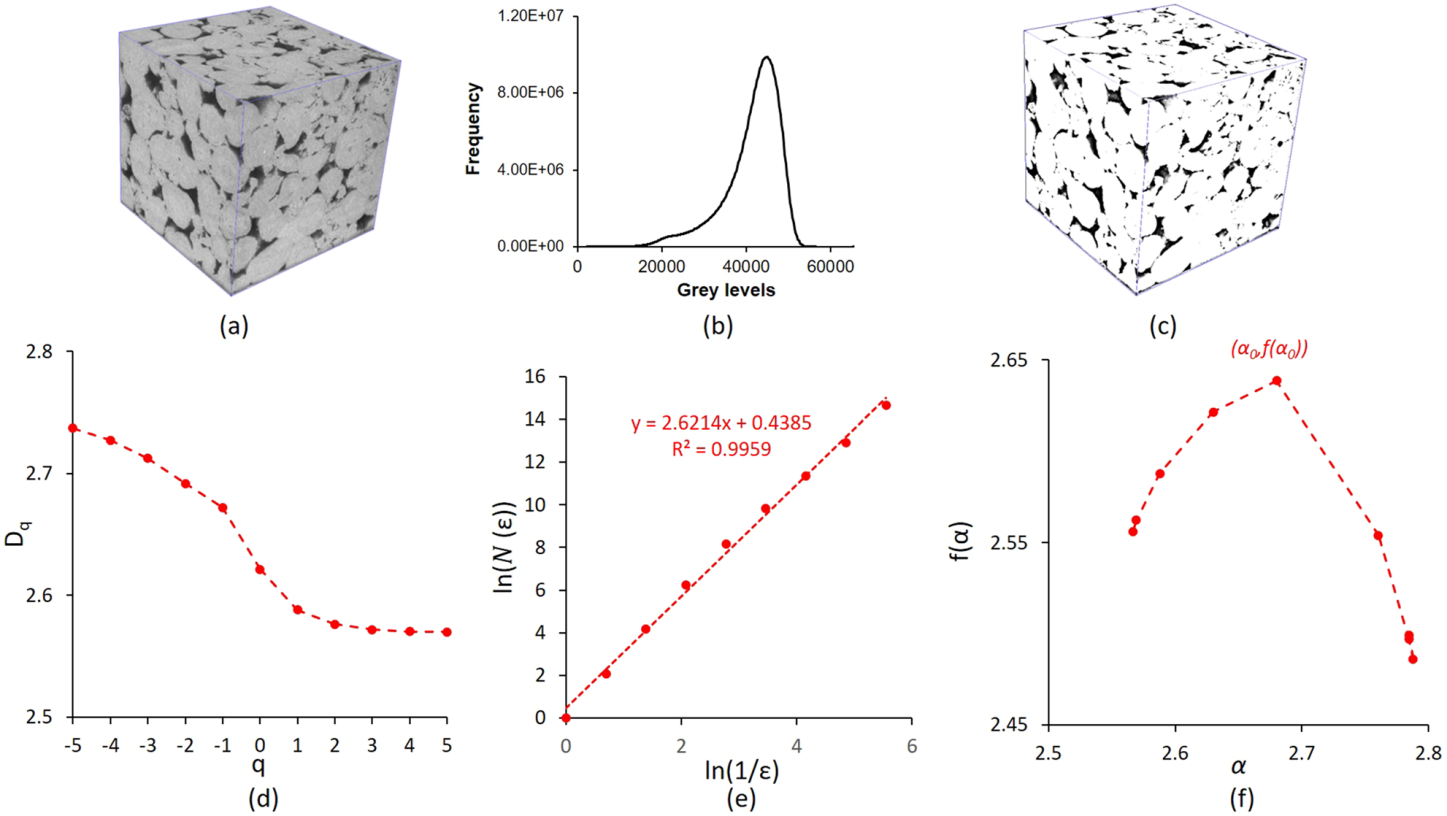
Fractal dimension calculation process based on 3D X-ray micro-computed tomography for sample S_1_. (**a**) The original 3D image, (**b**) histogram of grey levels with the thresholds implemented for 3D image segmentation, (**c**) segmented image, (**d**) the generalized dimension D_q_, (**e**) Fractal dimension D = 2.62 calculated as slope of the line ln(N(ε)) as a function of ln(1/ε), and (**f**) Singularity spectrum f(α) calculated from 3D segmented image.

In order to calculate the fractal and multifractal parameters from 3D X-Ray Digital images, the pore network needs to be identified using an image segmentation method^32^. Indeed, every generated voxel inside the three-dimensional X-ray image represents a grey level, coded in 16 bits, associated to the density at this precise spatial location. High and low grey level values denote solid and porous phases, respectively. Standard approaches implement thresholding algorithms to find automatically grey level limits separating these different phases in the image. In this study, we use a common thresholding method called the bi-level segmentation technique. This approach includes the grey levels spatial distribution into the histogram shape information to calculate thresholds separating porous and solid phases. The main advantage of this method is the use of region growing strategy to cope with the fuzzy transition zone representing the unresolved micro porous phase by X-ray imaging. Nevertheless, limitations in image acquisition resolution may lead to an intermediate mode in the histogram related to the presence of micro pores below image resolution. In this paper, the bi-level segmentation method was implemented to find automatically the two thresholds separating the three phases. Lower and higher thresholding grey level values for the sample S_1_ were estimated respectively as 28,253 and 33,023 (Fig. 8b). In order, to calculate the fractal dimension, the intermediate phase was not considered. Each voxel belonging to this intermediate phase represents mixture of pore and grains below image resolution where geometry cannot be captured. Therefore, the binary image used for fractal dimension estimation implemented the lower thresholding value.

#### Image fractal and multifractal parameters

The 3D Box-Counting method was implemented to analyse patterns of self-similarities in 3D segmented images by breaking them into a grid of boxes. This approach is used, in general, to approximate the Hausdorff dimension of a fractal dimension^45^. Boxes correspond to cubes used in investigating, by zooming in and out, the geometric complexity in a data at several length scales. In our application, pore structure was studied as main pattern of interest in three-dimensional binary images. The box-counting quantifies the presence of pores in each cube for a specific size of box. Practically, the number of cube boxes is calculated including the pore phase *N(ε)* for a specific cube side length *ε*. The procedure was repeated by covering the 3D image by a sequence of boxes of descending cube side lengths *ε*. Subsequently, the fractal dimension was estimated by the slope of the regression straight line representing the relationship between *ln(N(ε))* and *ln(1/ε)* as in the following equation:

11 $$\mathrm{D}=\underset{\varepsilon \to 0}{\mathrm{lim}}\frac{\mathrm{ln}(N\left(\epsilon \right))}{\mathrm{ln}(\frac{1}{\epsilon })}$$

The extension of the fractal theory is known as multifractal analysis, and it is used when the geometry of a system cannot be described by a single fractal dimension. Instead, a range of fractal dimensions is defined to fully define these objects. Multifractals are measured using a probability distribution *P*_*k*_ in each* k*^th^ box as ${P}_{k}(\upvarepsilon )\sim {\upvarepsilon }^{{\alpha }_{k}}$ as in shown in Eq. (3), where *ε* is the box size and ${\alpha }_{k}$ is the Lipschitz–Holder exponent characterizing the singularity strength in the *k*^th^ box. Zhang et al.^23^ proposed the use of ${\alpha }_{k}$ factor to measure the level of complexity in the spatial distribution. They suggested that the number of boxes, denoted as ${N}_{\alpha }(\varepsilon )$, can be used to represent the probability *P*_*k*_ of singularity strengths between *α* and α + d*α*. Additionally, the size of the boxes, *ε*, can be related to ${N}_{\alpha }(\varepsilon )$ as following ${N}_{\alpha }(\varepsilon )\sim$ ε^−f (α)^ where *α* corresponds to the singularity and *f*(*α*) is the singularity spectrum as in (4,9,10). Similarly, the mass exponent *τ*_*q*_, the generalized dimension *D*_*q*_, the partition function *χ(q,ε),* concentration of pore size distribution *α*_*0*_, the non-uniformity *Δα* = (*α*_*min*_-*α*_*max*_), the asymmetry degree in the vertical axis *Δf* = *f*(*α*_*min*_) − *f*(*α*_*max*_) and the asymmetry degree in the horizontal axis *R*_*d*_ of the range [*α*_*min*_*, α*_*max*_] with respect to *α*_*0*_ were derived from the same equations in section "Samples and experiments". Figure 8 illustrates the result of image segmentation in addition to the generalized dimensions *D*_*q*_ and *f*(*α*) the singularity spectrum obtained for sample S_1_.

#### Numerical simulations of rock properties

Simulating permeability at pore scale using Lattice Boltzmann (LB) method on 3D X-ray images involves the following steps: (1) acquisition of 3D images of a porous material, (2) segmentation of the images to extract pore network and solid matrix, (3) assignment of boundary conditions and fluid properties to the pore network, (4) simulation of fluid flow through the pore network, and (5) calculation of permeability from the simulated fluid flow. The method considers the micro-scale features of the porous material, providing a more accurate representation of permeability compared to macro-scale models. In this approach, the fluid is modelled as particles moving in a lattice structure and their movement is described using a time and space distribution function. The governing equation calculates the density *f(x,t)* of the particles at each iteration, taking into account both the streaming and colliding terms, where *x* represents the location and *t* represents the time as in the following equation:

12 $$f\left(x+{e}_{i}, t+1\right)=f\left(x, t\right)+\Omega \left(x,t,F,\tau ,{u}_{i}\right)$$

The particle movement direction in the lattice is indicated by *e*_*i*_, where *i* is the index for a specific direction. The particle velocity is *u*_*i*_, *τ* is the relaxation time controlling the rate of approach to equilibrium, *Ω* is a collision operator required to satisfy the conservation of total mass and total momentum, and *F* is an external force term. The LB method has several advantages as it calculates the particle velocity at each iteration using only the velocities of surrounding particles, making it easily implementable on high performance computer clusters. A multi-relaxation-time model based on the Lattice Bhatnagar–Gross–Krook (LBGK) scheme for the 3D lattice was used in this study, following a D3Q19 lattice model^47^. The flow was set to be periodic between the inlet and outlet and the bounce-back rule was used to manage solid–fluid boundaries. Additionally, a no-slip boundary condition was implemented at the solid–fluid interface. When the steady state is reached, the permeability is estimated using the unidirectional Darcy’s law as in the following equation:

13 $$K = \frac{\mu LQ}{A\Delta P}$$

where* K* is the absolute permeability, *ΔP* represents the pressure difference along the length of the sample, *A* is the surface section area, *Q* stands for the flow rate and µ is the dynamic fluid viscosity. The permeability value estimated is in lattice units and are converted to a real value using the resolution of the scanned image. Moreover, the porosity can be determined by calculating the ratio of the number of voxels within the segmented pore space to the total number of voxels in the image. This value is calculated directly from the segmented 3D images.

## Results and discussion

### Numerical and experimental fractal analysis of pore structures

**Figure 9:**
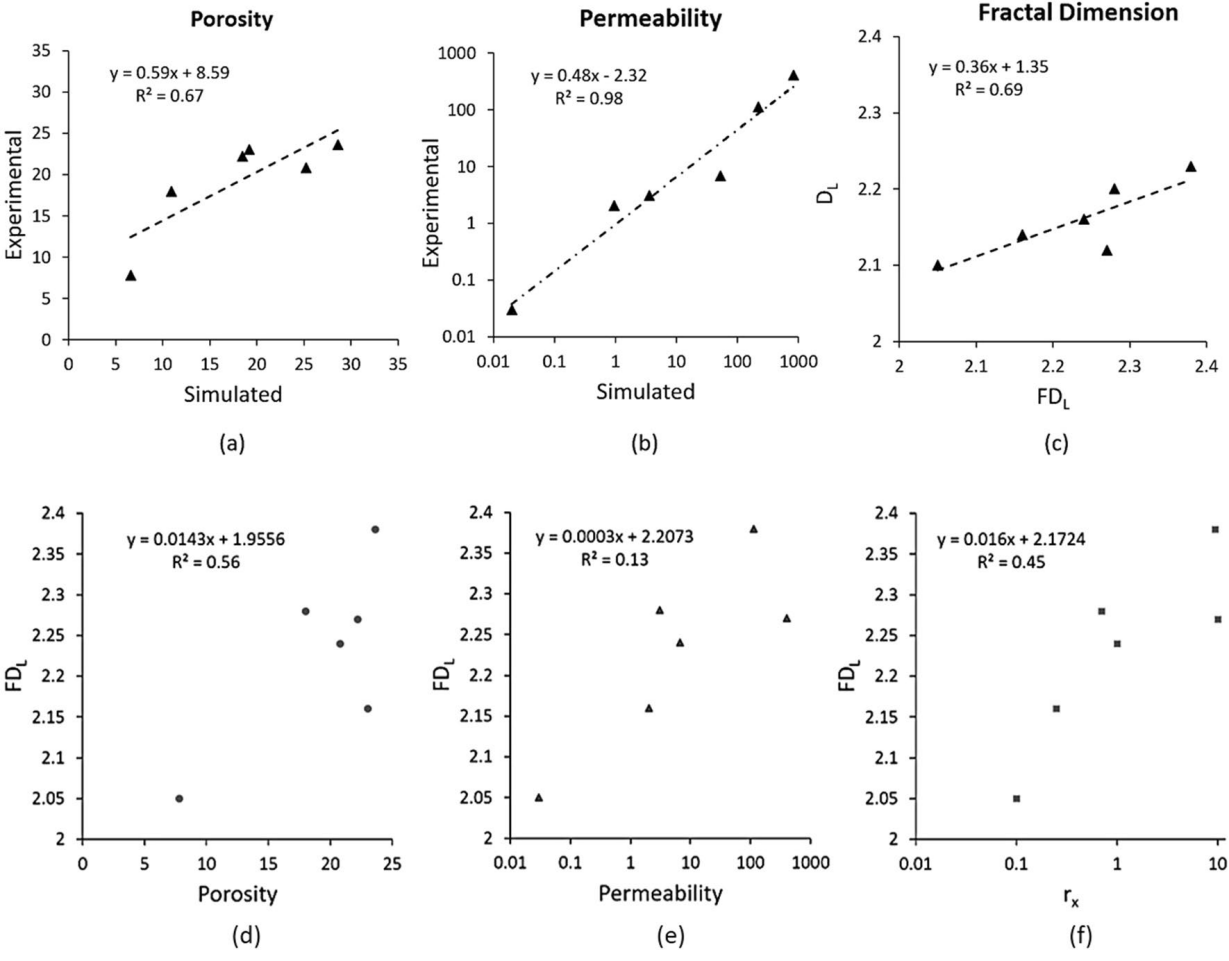
Correlation between experimental and simulated rock properties for the 6 samples: (**a**) porosity, (**b**) permeability, (**c**) experimental and simulated fractal dimensions *D*_*L*_ and *FD*_*L*_. Correlations between image fractal dimensions *FD*_*L*_ and (**d**) porosity, (**e**) permeability, and (**f**) Radius at Swanson’s segmentation point.

Comparison between the simulated and experimental properties showed a good agreement for both porosity and permeability rock properties with coefficient of determinations of R^2^ =  + 0.69 and R^2^ =  + 0.98, respectively (Fig. 9a,b). The correlation between experimental and simulated permeability values is higher than those of porosity because permeability have larger magnitude variation, from 0.02 to 800 mD. These observations indicate that the three-dimensional images captured successfully digital pore networks representative of the real pore space in subsets used for HMPI experiments. This result confirms that image segmentation and Lattice Boltzmann methods provide accurate results for porosity and permeability when implemented on high quality resolution images as reported in several studies^30,48,49^. Figure 9c reveals a fair agreement between the fractal dimension *D*_*L*_ derived from HPMI and the fractal dimension *FD*_*L*_ calculated form 3D images with a coefficient of determinations R^2^ =  + 0.69. This observation suggests the existence of a link between pore structures description using image fractals and experimental rock properties in the studied carbonate samples.

Furthermore, the image fractal dimension *FD*_*L*_ showed a positive correlation with porosity with a coefficient of determination R^2^ = 0.56 for a direct linear fitting relationship (Fig. 9d). This observation suggests that high porosities correspond to high image fractal dimension *FD*_*L*_ values because high porosity images usually have more compact pore structure. Figure 9e revealed no correlation (R^2^ =  + 0.13) between the image fractal dimension and the experimental permeability measurements obtained from HPMI. Indeed, the fractal dimension *FD*_*L*_ also called capacity dimension usually captures the average presence of pores rather than their connectivity^29^. Furthermore, a positive correlation was revealed between the fractal dimension *FD*_*L*_ and the radius at Swanson’s segmentation point with a relatively fair coefficient of determination R^2^ =  + 0.45 (Fig. 9f).

### Heterogeneity characterization using HPMI experiments

Various types of pores with varying sizes develop in carbonate reservoirs, leading to heterogeneity in the structure of the pores (Chen et al., 2016). A brief description of the six samples features and their limestone textures is provided in Table 1. The permeability of samples S_1_ and S_2_ derived from HPMI results analysis are 401.63 mD and 112.84 mD respectively (Table 3). These high values of permeability shows that samples are dominated by large pore throats. This observation is in agreement with the wide bimodal pore throat size distributions with a major peak at around 16 µm (Fig. 3). A second peak appears around 0.113 µm and 0.21 µm for samples S_1_ and S_2_, respectively. Both samples show well connected large pores in their macro-porous phases and 3D-MCT scan data, and the presence of inter-particle and intra-particle pores. In contrast, the samples S_3_ to S_6_ showed low permeability values spanning from 0.03 mD to 6.75 mD, suggesting that the samples are dominated by small pore throats (Table 3). Samples S_3_ and S_4_ showed bimodal pore throat size distributions, narrower than samples S_1_ and S_2_, with a major peak at around 1.2 µm (Fig. 3). A smaller peak appeared for both samples at 0.58 µm as well.

**Table 3:** Experimental and numerical porosity and permeability values for the six samples.

Samples	HMPI		Digital simulations	
	Porosity (φ(%))	Permeability (K(mD))	Porosity (φ(%))	Permeability (K(mD))
S_1_	22.2	401.63	18.5	833
S_2_	23.6	112.84	28.6	222
S_3_	20.8	6.75	25.2	52
S_4_	18.0	3.09	10.9	3.62
S_5_	23.0	2.03	19.2	0.95
S_6_	7.8	0.03	6.6	0.02

**Figure 10:**
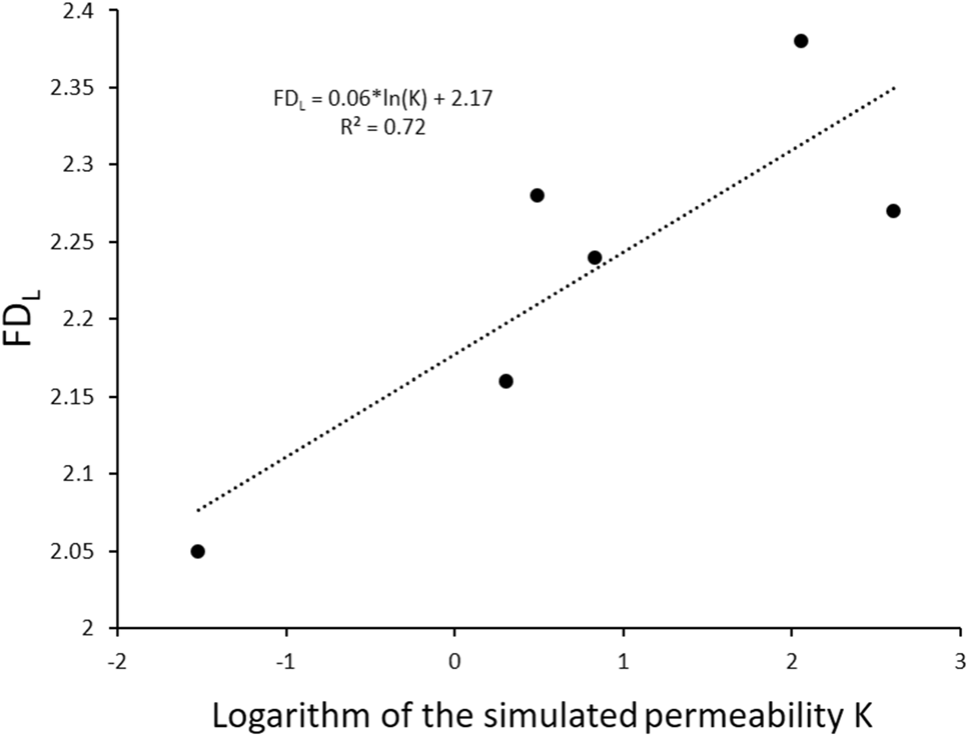
Relationship between fractal dimensions *FD*_*L*_ and simulated permeability log(*K*) for sample S_1_ to S_6_.

**Figure 11:**
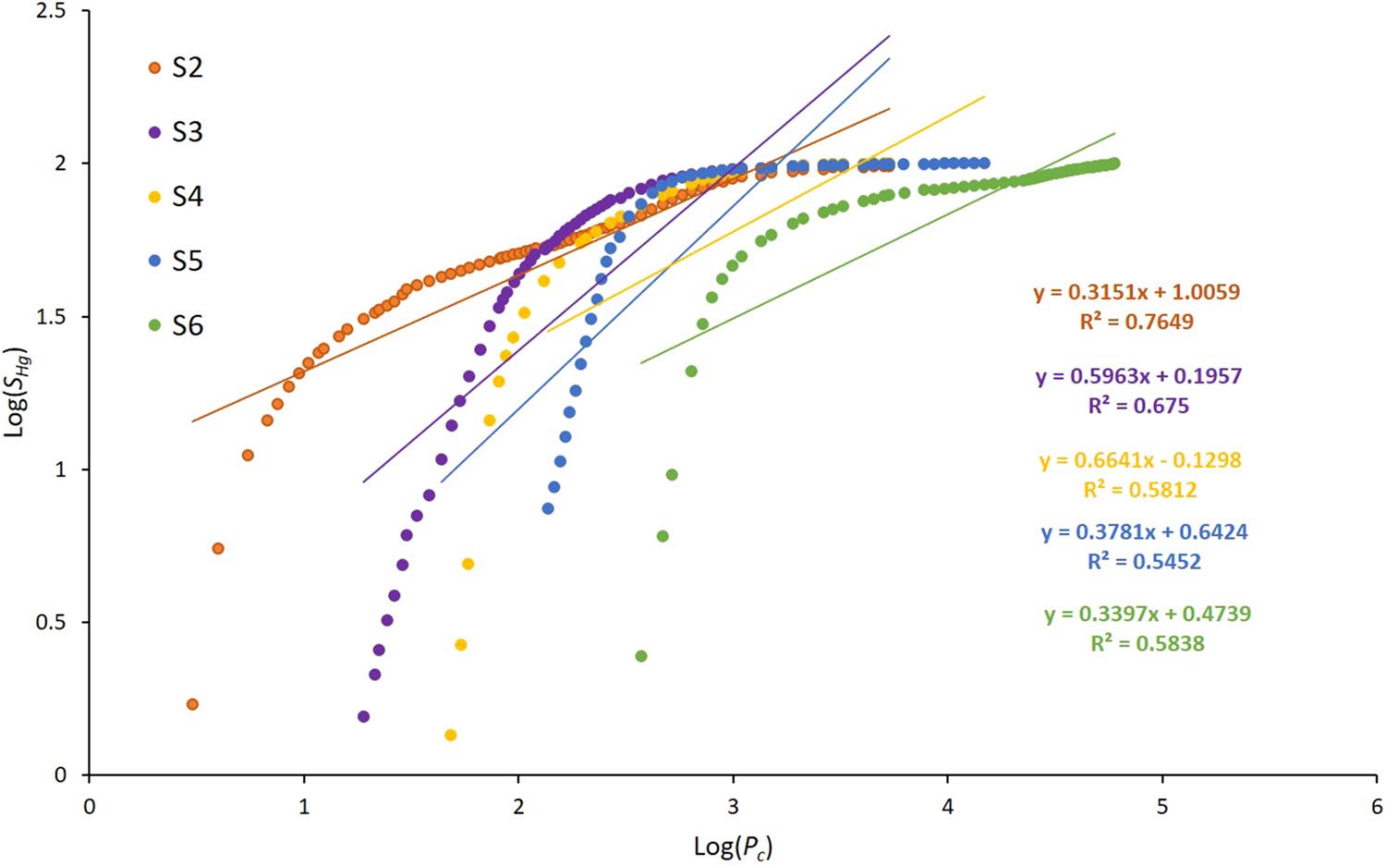
Log–log plot relating *S*_*Hg*_ the mercury saturation to the capillary pressure *P*_*c*_ for sample S_2_ to S_6_.

**Figure 12:**
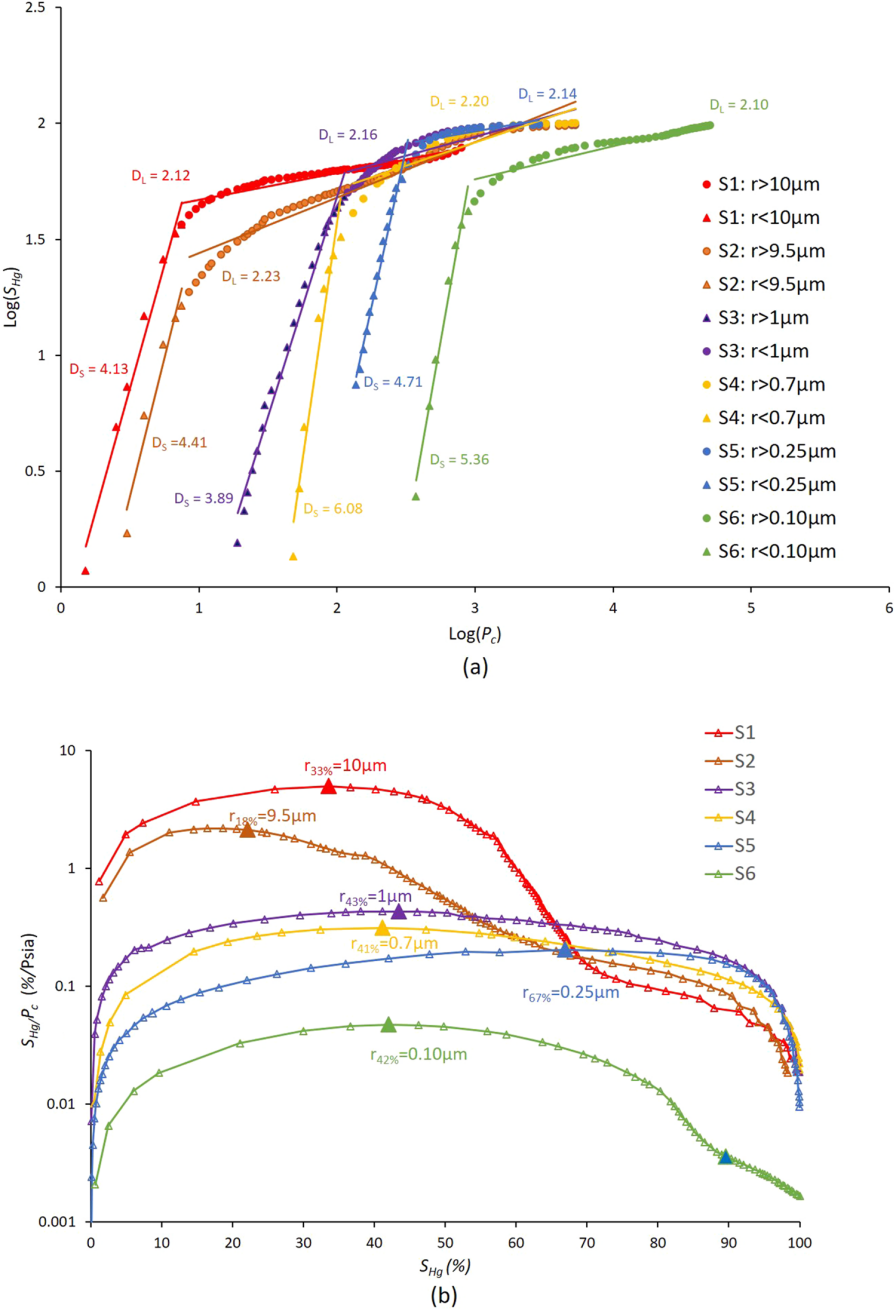
(**a**) Swanson’s segmentation points for samples S_1_ to S_6_, and (**b**) Log − log plot of *S*_*Hg*_ and *P*_*c*_ obtained from HPMI experimental measurements for samples S_1_ to S_6_ revealing double-fractal behaviour.

The 3D-MCT image of sample S_3_ acquired at 0.5 µm resolution showed a well-connected macropore system and presence of dolomite rhombs within the micrite matrix. The 3D-MCT scan of sample S_4_ at 0.5 µm resolution showed that the micro-porous phase exits inside the leached grains in grain supported texture. The HPMI data of samples S_5_ and S_6_ revealed unimodal pore throat size distributions with a major peak at 0.27 µm and 0.14 µm, respectively. Both samples display relatively narrow distributions by comparison to the other four samples (Fig. 3). The NCT image acquired at 60 ηm resolution revealed that most of the microporous phase exists inside the mud matrix for sample S_5_ and exists within the micrite between the oncoidial allochems for sample S_6_. Furthermore, the fractal characteristics of the pore space for the six samples were investigated using experimental HPMI measurements. In percolation theory suggests that the flow properties are predominantly influenced by a characteristic length, which plays a pivotal role in governing the fluid flow in reservoir rocks^42^. An increased fractal dimension indicates a shift in the regular pore morphology towards a more complex form^15,21,41^. Consequently, this transformation results in a decrease in both fluid flow and permeability. Several researchers have used fractal dimension on tight sandstone to predict permeability. For example, Lai and Wang^41^, applied Li's^21^ model to assess the fractal characteristics of tight sandstones, revealing the presence of two distinct fractal regions in all tight sandstone samples in their investigation. The fractal dimension value acts as a quantitative measure of reservoir heterogeneity, demonstrating that an elevation in the fractal dimension corresponds to a more complex pore structure, ultimately resulting in decreased permeability. Nevertheless, in our study Fig. 10 reveals the existence of a relatively strong direct linear relationship between the fractal dimension and the logarithm of the simulated permeability with R^2^ = 0.72. This outcome is consistent with the result reported by Xin et al.^26^ revealing an increase of permeability with respect to the increase of fractal dimension in a fractured-vuggy carbonate reservoir. However, Zhang et al.^15^ studied fractal dimension of pore structures of Lower Carboniferous carbonate reservoir and showed the existence of an inverse linear relationship with permeability (R^2^ = 0.75). Figure 11 shows the log − log plot of *S*_*Hg*_ and *P*_*c*_ samples S_2_ to S_6_. As for sample S_1_ the plot showed poor linear relationships fitting between the variables. Nevertheless, Fig. 12a revealed that all curves have a double-fractal behaviour characterized by the Swanson segmentation points. The curves obtained by plotting *S*_*Hg*_ versus *S*_*Hg*_/*P*_*c*_ denoted sharp apex for each sample (Fig. 12b). Table 4 summarizes the maxima values obtained for each sample at the corresponding saturation percentage *S*_*Hg*_.

**Table 4:** HPMI fractal dimensions, image fractal dimensions and Swanson’s segmentation points for the six samples where *x* represents the saturation of Hg in %.

Samples	HPMI fractal dimensions				Image fractal dimension				Segmentation point	
	*D*_*L*_	*R*^*2*^	*D*_*f*_	*R*^*2*^	*D*	*R*^*2*^	*FD*_*L*_	*R*^*2*^	*S*_*Hg*_ (%)	*r*_*x*_ (µm)
S_1_	2.12	0.95	2.24	0.53	2.62	0.99	2.27	0.99	33	10
S_2_	2.23	0.94	2.31	0.76	2.63	0.97	2.38	0.97	18	9.5
S_3_	2.16	0.90	2.59	0.67	2.53	0.98	2.24	0.98	43	1.0
S_4_	2.20	0.87	2.66	0.58	2.68	0.99	2.28	0.99	41	0.7
S_5_	2.14	0.88	2.37	0.54	2.74	0.98	2.16	0.98	67	0.25
S_6_	2.10	0.91	2.33	0.58	2.59	0.99	2.05	0.99	42	0.10

The double-fractal characteristic is revealed by piecewise linear regressions showing good fits with a positive coefficient of determination R^2^ spanning in the interval [0.87, 0.95] for the six samples. The fractal dimensions of the large pores, denoted *D*_*L*_, ranges from 2.04 to 2.23. In addition, a good fit is revealed for the left segments corresponding to smaller pores in Fig. 12a, the estimated fractal dimensions *D*_*S*_ values are all greater than 3.0, which contradicts the Euclidean dimension revealing absence of fractal characteristics. Among, our studied six samples, only sample S_2_ may reveal three sections for the log(Pc) versus log(Shg) curves. However, the estimation of the fractal dimension in the intermediary section provided also a fractal dimension larger than 3.0 contradicting the Euclidean dimension. Therefore, we used only *D*_*L*_ revealing the fractal behaviour for macro-pores.

**Figure 13:**
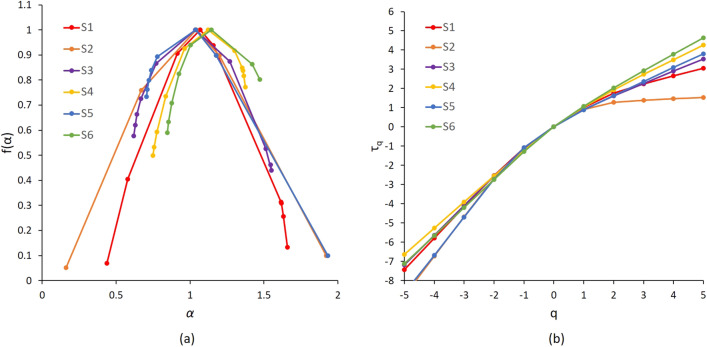
Multifractal parameters for samples S_1_ to S_6_ from HPMI experimental measurements: (**a**) singularity spectrum *f*(*α*), and (**b**) the mass exponent *τ*_*q*_.

Moreover, several multifractal parameters were calculated based on the HPMI measurements of the six samples. The mass exponent *τ*(*q*) and the singularity spectra *f*(*α*) were calculated for the six samples. Figure 13a illustrates slope variations of the mass exponent *τ* with respect to *q* for the six samples. Samples S_1_ and S_2_ show higher changes in slopes values compared to the other four samples. These large variations are due to the higher heterogeneity in pore throat size distributions in these two samples described in both HPMI data and observed in 3D X-Ray images. This observation confirms the ability of multifractal parameters to assess quantitatively heterogeneity in HPMI measurements. The singularity curves for the six samples revealed convex parabolic shape as illustrated in Fig. 13b. The samples S_1_, S_4_ and S_6_ revealed singularity spectra curves *f*(*α*) with a left asymmetry around *α* equals to 1. Singularity curves of samples S_1_, S_4_ and S_6_ showed wider left portions with sharper slopes than the right ones. However, asymmetric singularity curves of samples S_2_, S_3_ and S_5_ revealed wider right portions with sharper slopes than the left ones. These behaviours indicate that distributions of pores reveal a multifractal behaviour for all six samples. Moreover, singularity parameters *Δα* and *Δf*(*α*) were calculated to assess heterogeneities characteristics of the samples. Table 5 summarizes the results values for the six samples. The concentration of pore size distribution α_0_ values were in the range [1.03, 1.14] for the six samples. Smaller α_0_ values indicate larger concentrated distributions. The degree of heterogeneity in the distribution of probability measures of physical quantities over the entire fractal structure is indicated by the width of the multifractal spectra *Δα*. Samples S_1_ and S_2_ revealing strongest heterogeneity of pore distributions revealed largest Δα values among the six samples of 1.22 and 1.91, respectively. The range of non-uniformity degree *Δα* was [0.47, 0.93] for the other four samples. The most homogeneous sample in term of pore size distribution appears is sample S_5_ with the *Δα* value of 0.47. These results confirm the ability of this multifractal parameter to describe quantitatively the heterogeneity in samples. In addition, *Δf*(*α*) values were positive for samples S_1_, S_4_ and S_6_ indicating that a large probability subset was dominant in these samples. The samples S_2_, S_3_ and S_5_ show *Δf* values that are negative indicating that a small probability subset was dominant in these samples.

**Table 5:** Multifractal parameters from HPMI measurements.

Samples	*α*_*0*_	*α*_*min*_	*α*_*max*_	*Δα*	*Δf*	*R*_*d*_
S_1_	1.06	0.43	1.65	1.22	0.93	0.39
S_2_	1.04	0.06	2.06	1.99	0.94	− 0.04
S_3_	1.03	0.61	1.55	0.93	0.56	− 0.09
S_4_	1.12	0.75	1.37	0.62	0.50	− 0.60
S_5_	1.03	0.70	1.17	0.47	0.26	0.19
S_6_	1.14	0.84	1.47	0.62	1.14	− 0.02

### Multifractal characteristics of pore structures using image analysis

**Figure 14:**
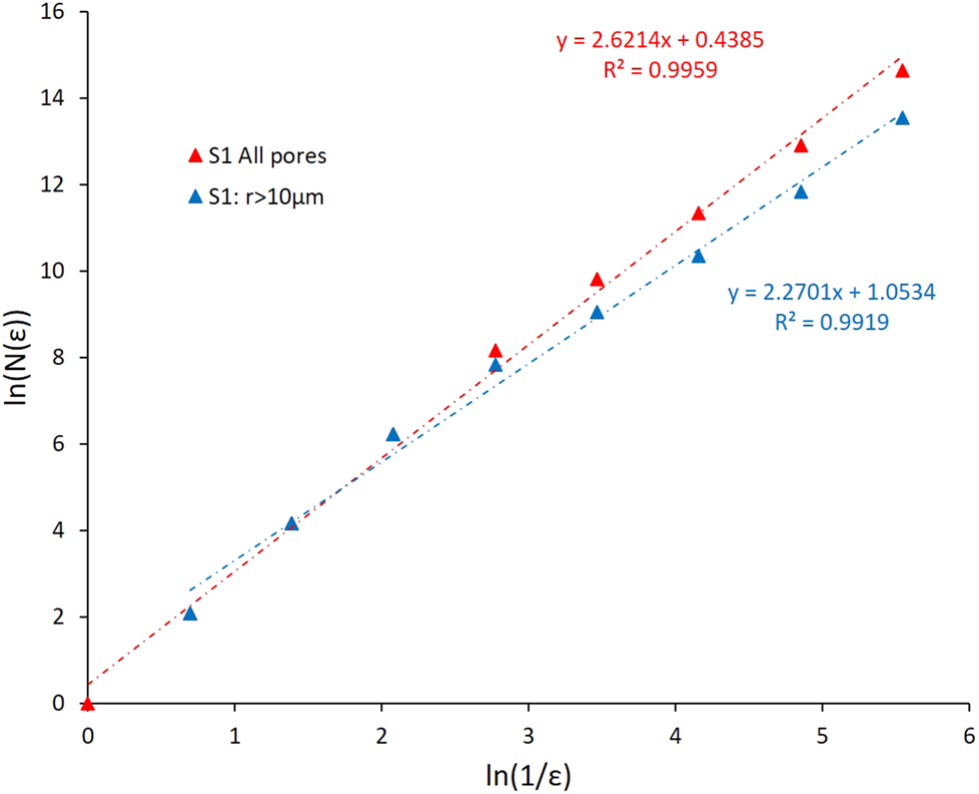
Fractal dimensions calculated as slope of the line ln(N(ε)) as a function of ln(1/ε) in sample S_1_: in red D = 2.62 including all pores and in blue D = 2.27 including all pores with r > 10 µm.

The analysis of 3D-MCT images of samples S_1_–S_4_ produced fractal dimensions values *D* ranging from 2.53 to 2.64, whereas NCT images gave fractal dimensions 2.59 and 2.74 for samples S_5_ and S_6_, respectively. The image fractal dimensions *D* values (Table 4) reveal larger values when compared to the range of experimental fractal dimension obtained from HPMI which are in the range [2.10, 2.23]. This result was expected as fractal dimension calculation was based on all pores included in images. Therefore, to have a more representative comparison, image fractal dimensions (*FD*_*L*_) were recalculated keeping only pores with sizes larger than the Swanson’s segmentation points. Figure 14 illustrates the comparison between fractal dimension values calculated including all pores in red and including only pores larger than 10 µm in blue. The recalculated fractal dimension values vary in the range [2.05, 2.38] (Table 4). The fractal scaling law, as observed in various studies, suggests that an increase in porous area (in 2D images) or volume (in 3D images) will result in higher fractal dimensions^23^. Consequently, the decrease of fractal dimension values is in agreement with results reported in these previous studies.

Furthermore, samples S_1_ and S_2_ revealed image fractal dimensions *FD*_*L*_ values of respectively 2.27 and 2.38 larger than the four other samples with values ranging in the interval [2.05, 2.28]. This observation indicates the effectiveness of fractal dimensions in capturing complexity in pore structures of samples through the imaged pore network.

**Figure 15:**
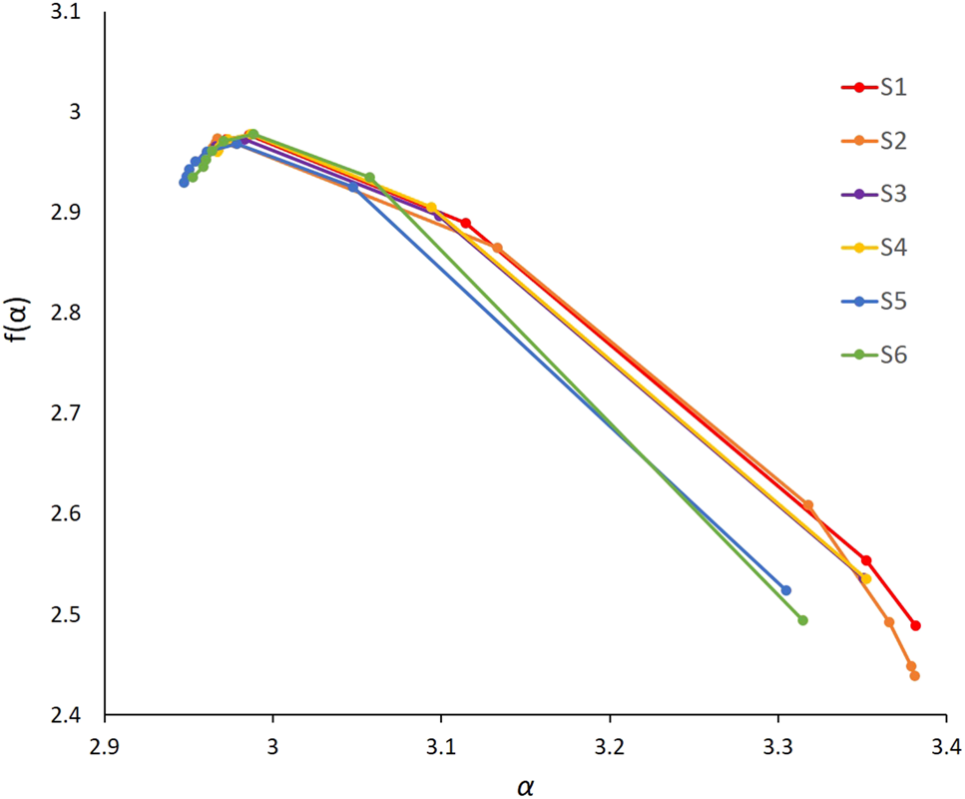
Singularity spectra f(α) for samples S_1_ to S_6_ calculated from 3D segmented images.

The singularity curves *f*(*α*) for the six samples showed right sided asymmetric convex parabolic shapes as illustrated in Fig. 15. Also, Table 6 summarizes the image multifractal parameters results for the six samples. The concentration of pore size distribution values α_0_ ranged between 2.977 and 2.988. Larger *α*_*0*_ values indicate smaller concentrated distributions. Samples S_1_ and S_2_ revealed largest Δα values among the six samples respectively 0.413 and 0.416 whereas *Δα* was in the range [0.357, 0.392] for the other four samples. This observation confirms that the non-uniformity degree of the pore structures *Δα* captures heterogeneity from 3-dimensional image data. Moreover, *Δf*(*α*) values were negative for the six samples indicating that a small probability subset was dominant in these samples.

**Table 6:** Multifractal parameters from 3D images.

Samples	*α*_*0*_	*α*_*min*_	*α*_*max*_	*Δα*	*Δf*	*R*_*d*_
S_1_	2.985	2.967	3.381	0.413	0.488	− 0.377
S_2_	2.977	2.964	3.381	0.416	0.530	− 0.391
S_3_	2.983	2.958	3.350	0.392	0.436	− 0.342
S_4_	2.986	2.967	3.352	0.385	0.442	− 0.346
S_5_	2.978	2.947	3.304	0.357	0.444	− 0.294
S_6_	2.988	2.952	3.314	0.362	0.484	− 0.289

### Correlation between HPMI and digital multifractal parameters

**Figure 16:**
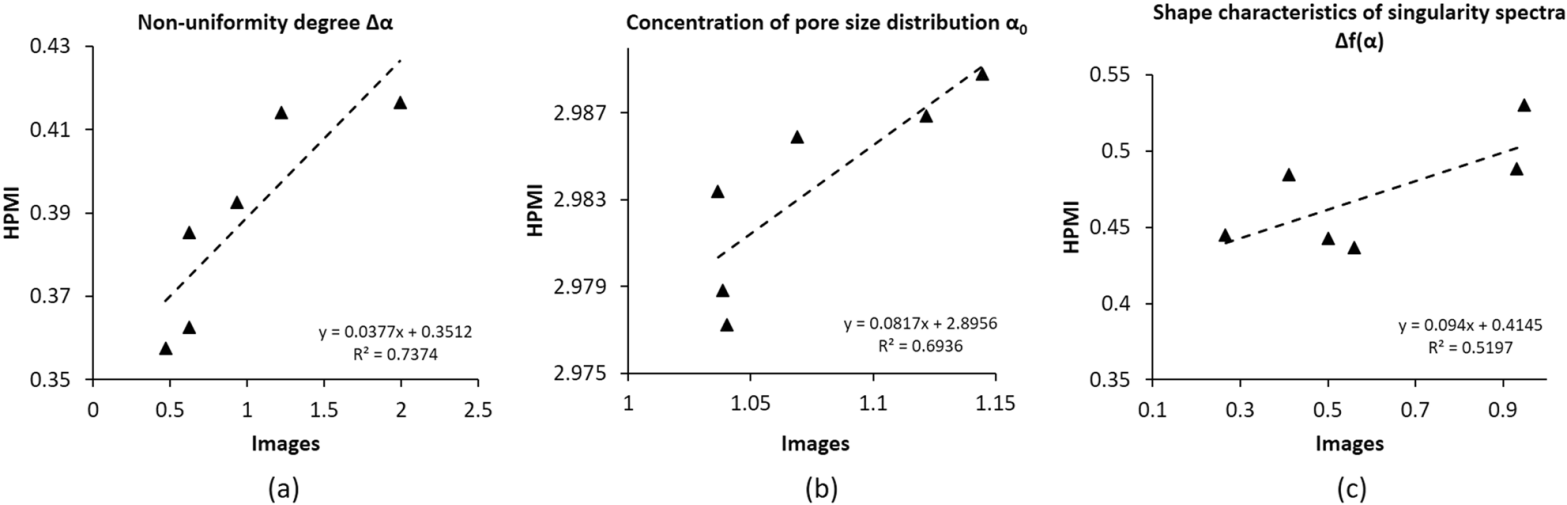
Correlation between experimental and digital multifractal parameters for samples S_1_ to S_6_: (**a**) non-uniformity Δα, (**b**) concentration of pore size distribution α_0_, and (**c**) shape characteristics of singularity spectra Δf(α).

The correlations between multifractal parameters derived from HPMI experimental measurements and three dimensional images were analysed. Figure 16a revealed the existence of a strong linear relationship between the non-uniformity degree Δα calculated from HPMI and images with a relatively high determination coefficient R^2^ =  + 0.73. Furthermore, the concentration of pore size distribution *α*_*0*_ showed also a relatively good linear relationship with a determination coefficient R^2^ =  + 0.69 (Fig. 16b). However, the relationship between the shape characteristics of singularity spectrums Δf(α) derived from images and HPMI experiments showed a weaker correlation with a determination coefficient R^2^ =  + 0.51 (Fig. 16c). These results show that the multifractality notion could describe independently both digital and experimental representations of the same data and that a correlation exists between these two representations.

The results obtained in this study indicate the relevance of multifractal parameters to describe heterogeneity quantitatively in pore structures from both experimental and digital data. The comparison between multifractal parameters ranges allowed discriminating homogeneous from heterogeneous samples. Nevertheless, this method suffers of lack of clear established reference ranges.

In future studies, it is imperative to explore a broader spectrum of experimental and digital measurements to establish statistically more comprehensive parameter ranges for Δα, α_0_, R_d_, and Δf(α) in carbonate rocks. These parameters serve as quantitative indicators for classifying the degree of heterogeneity in carbonate formations. Subsequently, in a secondary phase of the research, the goal is to identify the most significant multifractal parameters among these, influencing key rock properties such as porosity, permeability, and elasticity. Ultimately, our aim will be to integrate the most influential parameters among Δα, Δf(α), R_d_, and α_0_ as quantitative heterogeneity indicators into rock physics models for carbonate formations.

## Conclusions

The main findings of this study on pore structures investigation of the Lower Cretaceous, shallow marine limestone reservoir by HPMI and 3D X-Ray MCT and NCT in combination with fractal and multifractal theories are listed as below (Supplementary Information S1):

- The primary advantage of this study is to offer a quantitative tool for categorizing rock samples based on their heterogeneity within this reservoir. Indeed, multifractal analysis can effectively capture the heterogeneity present in carbonate samples, whether derived from experimental data or digital rock images. Furthermore, we establish the presence of a correlation between these multifractal parameters, even when estimated at various scales. These observations should be investigated for more samples in future studies to have statistically more representative and accurate results.
- The image simulated rock properties agree with experimental measurements suggesting that 3D imaging, and simulation are effective methods to represent HPMI experimental measurements. Image fractal dimensions values span from 2.05 to 2.38 and revealed fair correlation with experimental HPMI measurement of porosity. Overall, a good correlation was revealed with pore throat radius at Swanson segmentation point.
- Higher correlation between experimental and simulated permeability than porosity values due to larger magnitude of permeability variation indicates that 3-dimensional images captured successfully digital pore networks representative of the real pore space in subsets used for HMPI experiments.
- A double fractal behaviour was revealed in the curves relating mercury saturation to capillary pressure. The intervals with fractal characteristics are characterized by Swanson segmentation points. Fractal dimensions calculated from HPMI measurements showed a good correlation with experimental HPMI measurement of porosity.
- The existence of good correlation between image and experimental multifractal parameters suggest that the description of pore scale morphologies can be described accurately by 3-D X-ray images representing a foundation for further research.

### Supplementary Information

Supplementary Information.

## Data Availability

All data generated or analysed during this study are included in this published article [and its supplementary information files].
